# The Obeid-coronal malalignment classification is reliable and helps guiding decision-making and surgical management of adult spinal deformities: letter to the editor of BMC Musculoskeletal Disorders

**DOI:** 10.1186/s12891-023-06257-5

**Published:** 2023-02-23

**Authors:** Anouar Bourghli, Louis Boissière, Ibrahim Obeid

**Affiliations:** 1 Spine surgery department, King Faisal Special Hospital and Research Center, P.O.Box 3354, 11211 Riyadh, Saudi Arabia; 2 Clinique du Dos, Elsan Jean Villar Private hospital, Bordeaux, France

**Keywords:** Coronal malalignment, Obeid classification, Spinal deformity

## Abstract

A recently published article by Zhang et al. in BMC Musculoskeletal Disorders reported that the classification of coronal deformity based on preoperative global coronal malalignment for adult spinal deformity is questionable. The aim of the paper was to specifically discredit the Obeid-Coronal Malalignment (O-CM) classification. In this correspondence, we thought it judicious to clarify misunderstood concepts by the authors. We highlight several limitations of their study, and explain the deep interest of the classification from our perspective in order to avoid misleading the readers. Overarching, we aim to help the colleagues through a constructive rather than destructive approach to better understand the foundations of a coronal malalignment classification.

## Main text

We read with interest the article by Zhang et al. [1], which reported that the classification of coronal deformity based on preoperative global coronal malalignment (GCM) [2] for adult spinal deformity is questionable.

We appreciate the author’s effort to highlight their concerns regarding previously published classifications on coronal alignment. However, we have great concerns regarding the method that was used, which appeared to be for the sole purpose of discrediting an already established classification. It was in fact clearly stated at the end of the authors’ introduction that the purpose of their study was to “evaluate the rationality of classification of coronal deformity based on preoperative GCM by Obeid et al”. Such a study aim seems to be rather uncommon not only because of its formulation, but also because logically if a spinal classification is truly irrational, it will spontaneously not be adopted by the community of physicians and will be overpassed quickly by other, better classifications. As such, there is little need for a counter-study to try to discredit it on the pretense it will help guide the spinal surgeons that were supposedly mislead.

When assessing the alignment of the spine in the coronal plane, and in order to be as accurate as possible, we need to take into account the intraobserver and interobserver variability of measurement [3]. This is related to the position of the patient during the static X-ray that is performed in a standing position. In addition, trigonometry teaches us that 2°of pelvic obliquity gives 17 mm of Coronal Malalignment (CM) in a trunk of 50 cm [4]. Therefore, it seems necessary to establish a threshold value of malalignment in the coronal plane to account for the previously mentioned variability. Such threshold was defined as a deviation (to the right or the left side) of 20 mm (2 cm) of the C7 or T1 plumbline from the midline. This limit would be the minimum cut-off to respect the aforementioned prerequisites. Such geometrical justification had not been previously published.

Following the previously explained logic, the first paper by Obeid et al. [2] described the different types of coronal malalignment where type 0 (CM < 2 cm) is considered a coronally aligned spine. Indeed, any classification dealing with global alignment would need to define a normality status, for which many patients may not need surgery or would need a localized treatment. Therefore, when the head is immediately above the pelvis, this is clinically and radiologically an aligned spine in the coronal plane. However, it was clearly explained that such aligned status (type 0) did not mean that the patient would not need surgery or would not have specific coronal patterns. In fact, a complete paragraph on how to avoid postoperative coronal malalignment in a pre-operatively aligned spine was described and illustrated by a case, and it explicitly warned the reader and reminded them in the conclusion and summary algorithm that “a type 0 may potentially become a type 2 after surgical correction”. The paper [2] subsequently developed the different patterns (type 1 and 2) with their subtypes which were thoroughly detailed and illustrated. It should have been obvious, given the details that were explained inside the initial paper, that the patterns represented the keystone of the classification and its algorithm, not the global alignment based on the coronal plumbline.

The statement in the introduction of the authors’ paper that it is unknown whether the Obeid-Coronal Malalignment (O-CM) classification is feasible or not is not true because after the initial paper was published, a validation study for the classification involving 15 spine deformity experts was conducted which showed adequate intra and inter-rater reliability with a Fleiss kappa coefficient reaching a value of 0.9 for main curve types and 0.75 for subtypes with first modifier [5]. This finding emphasizes the fact that the main interest of the classification was to individualize patterns of coronal malalignment and verify their understanding and recognition by spinal surgeons, which was demonstrated. Another study followed which showed that CM distribution according to the Obeid-CM modifiers is age-dependent and that there is a correlation between CM types and Personal Related Outcome Measures with self-image and satisfaction being typically affected in the mobile spine [6]. Again, this outcome highlighted the importance of the patterns inside the classification. In addition, other studies are currently being conducted in order to further show the interest of the O-CM classification and its true practicality.

Identifying the patterns of coronal malalignment requires a deviation of the C7 or T1 plumbline in order to correctly recognize the main coronal curve and define whether the shift is toward the concave or the convex side. But, as stated earlier, the threshold of GCM in the paper by Obeid et al. [2] defined at 20 mm is far from the 30 mm [7] or 40 mm [8] that was used in other papers. Such small threshold, set for trigonometric reasons, took into account the variability of measurement that may occur on a static X-ray. It would be therefore difficult to establish subtypes of the 2 main patterns, as suggested in the authors’ method, because the margin of error for categorization would become important in order to truly differentiate, for example, a patient with a type 2 and 18 mm of deviation and another one with a type 2 and 21 mm of deviation. This would question the foundations of the method used by the authors, rendering the subsequent results doubtful and misleading.

We believe that type 0–1 and type 0–2, as defined by the authors, may both lead to a type 2 postoperatively in case of major curve overcorrection. This was in fact already explained in the first paper by Obeid et al., however we could not find such analysis showing the eventual conversion from one type preoperatively to another type postoperatively in the authors’ study, which is surprising. In addition, their corrective surgical strategy was not detailed in terms of areas of interest, types of correction gesture and levels of instrumentation according to the different patterns. Given the fact that they did not follow the treatment algorithm defined in the O-CM classification, the statement that it is not useful or that following it would lead to complications cannot be established.

It should be noted that the majority of the patients with CM that would require surgery show a deviation of their coronal plumbline to one of the sides (right or left), which is impacting their quality of life as demonstrated by several studies directly relating the GCM to functional scores [9, 10]. This underlines the importance of GCM and also weakens the authors’ study where no functional assessment was done at all as acknowledged in their limitations section. It was in fact stated that due to the retrospective feature of the study, functional evaluation was not performed. However, nowadays it is considered unreasonable for a clinical study to not include functional outcomes based on specific scores since the functional status of the patient is the finality of our management. Relying only on X-rays is not scientifically acceptable even in retrospective studies, especially in spinal deformities.

For all the previously mentioned reasons, we do not believe that the study by Zhang et al. [1] questioning the foundations of the O-CM classification [2] or even the Bao classification [11] as mentioned in their discussion, would make the aforementioned classifications doubtful. We rather believe that showing the importance of preoperative patterns adds important credit to the O-CM classification as it is the only one that detailed the patterns of coronal malalignment reaching a total of 6 subtypes in order to include the most common scenarios a surgeon could encounter and help them in categorization and management.

We again thank the authors’ effort in presenting their findings. But in our view, and from a global perspective, we do not believe that any new classification would pretend to be exhaustive, and on the contrary we see it as one of the many building blocks that would eventually improve our understanding of the spine science. Therefore, it may remain incomplete from a certain point of view, which is what usually represents the strongest motivator to continue elaborating productive research in our specialty. However, we do not believe that trying to demonstrate if a new classification is questionable or not is a very constructive approach, we would prefer to see instead a better classification scientifically demonstrating its superiority and reliability published, and leave the decision of using one or another to the spine community.

## Data Availability

Not applicable.

## Abbreviations

GCM: Global coronal malalignment
CM: Coronal malalignment
O-CM: Obeid-Coronal Malalignment
